# Parent–Child Interactions in Context: A Comparison of OCD and Non-clinical Families

**DOI:** 10.1007/s10578-025-01823-5

**Published:** 2025-03-15

**Authors:** Chloe A. McGrath, Maree J. Abbott, Sharlene C. Mantz, Margot O’Brien, David J. Hawes, Felicity A. Waters

**Affiliations:** 1 Clinical Psychology Unit, School of Psychology, The University of Sydney, Sydney, NSW 2006 Australia; 2 Rivendell Child and Adolescent Unit, Sydney Local Health District, Thomas Walker Estate, Hospital Road, Concord West, NSW 2138 Australia

**Keywords:** Obsessive compulsive disorder, Child and adolescent, Treatment, Cognitive behavioral therapy, Parent–child interaction

## Abstract

We investigated the context role of family factors in obsessive compulsive disorder (OCD) by comparing observed parent–child interaction behaviors of OCD and non-clinical families during three parent–child discussion tasks. We examined whether behaviors differentiating groups would be consistent across all discussions, or present only during specific tasks. We also investigated the effect of family-based cognitive behavior therapy on parent–child interactions and aimed to identify interaction behaviors associated with OCD symptom change. Thirty-nine children and adolescents (9–16 years old) and their parents participated in three discussion tasks about: (1) pleasant, (2) anxiety-provoking, and (3) conflict situations. Groups were compared on observer-rated parent and child behaviors. OCD dyads participated in discussions again after treatment. Results supported task-specific hypotheses for parent–child interactions in OCD, except for child warmth, demonstrating effects in the expected direction across all discussions. OCD and control families were distinguished on both child and parent behaviors, particularly during the anxiety discussion. Children and adolescents with OCD showed less warmth, confidence, positive problem-solving, and responsibility, and more doubt compared to controls. OCD group parents showed less warmth, confidence, and positive problem-solving, and more doubt and enhanced responsibility than controls. Treatment effects were present for the anxiety discussion exclusively. Pre-post reduction in OCD symptom severity was significantly correlated with an increase in child responsibility, likely related to a reduction in unhelpful avoidance behaviors targeted in treatment. Outcomes support models of OCD maintenance highlighting the importance of family environment factors in OCD symptoms, suggesting that parent–child interaction behaviors characterizing OCD families are contextual.

Obsessive compulsive disorder (OCD) is a debilitating mental health condition affecting 1–4% of children and adolescents [1–4]. The condition, characterized by obsessions and compulsions, not only significantly impacts the young person, but the whole family is also adversely affected [5–7]. Compulsions, or repetitive behaviors, are completed in an effort to neutralize obsessions, typically recurrent, intrusive thoughts or images, and the associated distress [5]. In addition to impacting family relationships and home life, OCD can also impair functioning across a range of other domains, including academic and social settings, inhibiting important child and adolescent development [8, 9].

Research has highlighted genetic risk factors and biological foundations of OCD; however accumulating evidence suggests that environmental factors also play an important role in the development and maintenance of the disorder. Cognitive behavioral models hypothesize that social learning contributes to the development of OCD [10]. During childhood and adolescence, the family environment plays a critical role in this social learning [10]. Recent findings have provided increasing support for the influence of family environment factors on the nature and course of child OCD, particularly in relation to neutralizing responses that help decrease the young person’s distress in the short-term (e.g., [11–15]). Some of the relevant family factors identified to date include family accommodation (FA), parental overinvolvement, criticism/blame, reduced warmth, and enhanced responsibility placed on the child. However, measurement of these family factors and our understanding of their function in the development and maintenance of OCD remains limited [16].

Family accommodation (FA), characterized by involvement of family member/s in an individual’s OCD symptoms, has been the most extensively researched family factor to date [6, 12, 17, 18]. Rates of FA are high in child OCD, with most families engaging in some accommodation behavior daily, such as providing reassurance or waiting for a child to complete compulsions [12, 17]. Although typically well-intentioned, usually to reduce child distress, in the longer-term FA maintains avoidance, anxiety, and OCD symptoms [11, 13]. FA has been significantly correlated with OCD symptom severity and child functional impairment in a range of settings [14, 15, 19–21].

Less is known about additional family factors associated with OCD in children and adolescents. Parental control/overprotection and rejection/criticism (in contrast to warmth and acceptance) have been associated with childhood anxiety disorders in general [22]. High levels of control/overinvolvement and criticism (or reduced warmth) have also been linked to child OCD more specifically. A study by Hibbs et al. [23] found that OCD families demonstrated higher levels of parental overinvolvement and criticism during a parent speech task compared to non-clinical families. Moreover, Valleni-Basile et al. [3] found that adolescents with OCD reported lower levels of warmth, emotional support, and closeness in their families compared with non-clinical adolescents. Other studies have highlighted significant correlations between high levels of criticism or blame in OCD families and increased OCD severity in children and adolescents [24, 25]. Parental enhancement of child responsibility is another factor associated with child OCD [26]. Cognitive behavioral models of OCD have identified inflated child responsibility as one of the core cognitive processes involved in development and maintenance of the disorder [27, 28]. One of the proposed pathways for development of inflated responsibility beliefs is through the family environment. In support of this assertion, Farrell et al. [26] showed that during a problem-solving discussion, parents placed significantly greater levels of responsibility on their child to solve the problem in OCD compared to non-clinical families.

Family environment factors, such as FA and criticism/blame, have also been linked to OCD treatment response. Children and adolescents from families with less FA prior to treatment have demonstrated improved treatment outcomes across a range of conditions, including gold-standard treatment for OCD, that is, cognitive behavioral therapy (CBT) with exposure and response prevention (ERP; [29, 30]). There is also some preliminary data to suggest that FA may mediate child and adolescent OCD symptoms [31–33], including a controlled trial by Piacentini et al. [33] showing that FA reduction temporally preceded OCD symptom improvement. Criticism/blame has also been implicated in treatment response. High levels of pre-treatment criticism and blame significantly predicted poor response to CBT for children and adolescents with OCD [34]. Another study showed that children and adolescents from families with lower levels of pre-treatment blame and conflict, and higher levels of family cohesion, had a 93% response rate to CBT compared to a 10% treatment response rate for those with poorer functioning in these three domains [35]. Less is known about the relationship between other family factors and young people’s response to OCD treatment.

Self-report data have typically been used to investigate the relationship between family factors and anxiety (including OCD) in children and adolescents. These data have predominantly included adults’ retrospective self-report of childhood family environment, although have also included parent and child self-report of current family environment (e.g., [36, 37]). Limitations of self-report methods to investigate family behaviors include potential unreliability of retrospective accounts of parenting behavior as well as response bias, particularly for undesirable behaviors, such as criticism or overinvolvement [37–39]. Increasingly, observational methods are being recommended and used to investigate the association between anxiety and family factors in an effort to measure these family constructs more precisely and objectively [37]. Recommendations for observational studies include the use of sub-categories for family dimensions assessed individually on a unidirectional scale, rather than the use of broader constructs and continuums that can underestimate the relationship between family factors and anxiety disorders [37, 40]. The broad dimension of “rejection”, for example, can be broken down into the sub-constructs: warmth, withdrawal, and aversiveness [37]. Similarly, “control” can be divided into the constructs of overinvolvement and autonomy granting [37].

Only a handful of observational studies have investigated the relationship between family factors and OCD in children and adolescents (e.g., [26, 41–44]). These few empirical studies have examined parent and child behaviors observed during 5-min parent–child discussion tasks with the aim of solving a specified problem. Barrett et al. [41] included discussions with topics relating to physical and social threats, respectively. Farrell et al. [26] and Mathieu et al. [43] included a discussion about an aversive situation, and Mantz and Abbott [42] and Schlup et al. [44] included a discussion about a “hot topic” (a problem in the family identified by the child or parent). Findings from these observational studies suggest that OCD families can be differentiated from control families, however, findings have been varied. It is also worth noting that inconsistencies in findings may be related to slight variations in task design, assessment methods, and measurement scales used.

Barrett et al. [41] found that OCD group parents demonstrated reduced confidence in their child’s ability, were less rewarding of their independence, and showed less positive problem-solving compared to clinical and non-clinical control parents (as well as less warmth when compared to non-clinical parents). Children and adolescents with OCD also demonstrated less confidence, positive problem-solving, and warmth, compared to controls. Unlike Barrett et al. [41], Farrell et al. [26] found no significant group differences in child or parent behavior (with the exception of parent responsibility enhancement). However, Farrell et al. [26] used bi-directional rating scales with opposing constructs on a continuum compared to the recommended unidirectional scales, as used by Barrett et al. [41], Mathieu et al. [43], and Mantz and Abbott [42]. Regarding responsibility enhancement, Farrell et al. [26] found that OCD group mothers enhanced their child’s responsibility during the problem-solving task more than their own, and more than non-clinical mothers. Mathieu et al. [43] found that OCD and non-clinical control groups could be differentiated based on parent aversiveness (OCD mothers showed more aversiveness), but not on the other behavioral dimensions of warmth, autonomy granting, withdrawal, and overinvolvement. In the Mathieu et al. [43] study, children and adolescents with OCD showed less warmth and confidence (approaching significance), and more withdrawal, compared to controls. Unlike Farrell et al. [26], Mathieu et al. [43] found no significant differences in responsibility enhancement between groups: mothers in both groups enhanced their child’s responsibility more than their own. Consistent with some of the previous findings (e.g., [41, 43]), Mantz and Abbott [42] found that children and adolescents with OCD demonstrated reduced warmth and confidence, and elevated doubt and withdrawal compared to controls, although Mantz and Abbott [42] found no significant group differences for parent behavior.

Only one child/adolescent OCD study published to date (i.e., [44]) compared observed behavior from a parent–child discussion task pre- and post-treatment. Treatment involved group CBT for children and adolescents with OCD with parent skills training. Schlup et al. [44] reported general improvement in both mother and child behaviors (e.g., criticism, overinvolvement, warmth, problem-solving) at post-treatment compared to waitlist, with the exception of mother avoidance and child doubt. However, no significant relationship was identified between change in mother or child observed behavior and OCD symptoms as a function of treatment.

The purpose of the current study was to enhance understanding of family factors relevant to child and adolescent OCD, and its treatment, using observational methods. The study aimed to examine the effect of context on observed parent–child interactions by uniquely comparing OCD and non-clinical families across three parent–child discussion tasks about (a) pleasant, (b) anxiety-provoking, and (c) conflict, situations. This design extends previous observational studies by distinguishing between anxiety-provoking and conflict situations, either not included, or combined, in previous studies (e.g., discussion tasks using “hot topics”; [42, 44]), and includes a pleasant situation condition to enable further comparisons. Further, the current study aimed to investigate whether parent and/or child behaviors distinguishing groups were observed consistently across the three discussion tasks or were present only during a particular discussion (e.g., the anxiety-provoking situation discussion). The present study also compared parent–child behaviors, observed during the three tasks, pre- and post-treatment for the OCD families completing FCBT for OCD [45, 46], further strengthening the study’s design. As such, the current study aimed to identify observed parent–child behaviors associated with OCD symptom change. This is the first child/adolescent OCD study to compare behaviors observed during a parent–child interaction task pre- and post-treatment for participants completing individual (rather than group) treatment for OCD. A further strength, in line with recommendations (e.g., [37]), was the use of unidirectional scales to individually assess sub-constructs, such as overinvolvement and autonomy granting, in order to measure these family constructs more precisely. The current study tested three main sets of hypotheses:

1. Based on previous findings (e.g., [26, 41–43]), while being mindful of the mixed nature of these findings, we hypothesized that OCD group children and adolescents and their parents would score higher on negative dimensions (e.g., aversiveness, withdrawal, doubt, reassurance seeking/providing, and overinvolvement), and lower on positive dimensions (e.g., warmth, confidence, positive problem-solving, and autonomy granting), compared to controls. Based on preliminary findings from a previous study (i.e., [26]), we predicted that OCD dyads would enhance child rather than parent responsibility, and more so than controls.
2. Two competing exploratory hypotheses included whether differences between OCD and control groups in observed parent–child behavior would be (a) consistent across the three discussions, or (b) present only in the discussion task/s that evoke higher stress responses, in particular the anxiety discussion.
3. We anticipated that the individual FCBT used in the present study, directly targeting a number of family factors, would result in significant pre- to post-treatment improvements in OCD dyad’s interaction behaviors, in particular behavior likely to be addressed during treatment (directly or indirectly), such as overinvolvement, autonomy granting, aversiveness, confidence, doubt, reassurance seeking/providing, problem-solving, and responsibility enhancement. While taking into account the non-significant findings by Schlup et al. [44], we predicted significant correlations between OCD symptom change and family factor change for the above-mentioned behavioral dimensions in response to individual FCBT targeting a range of family factors, compared to group CBT utilized by Schlup et al. [44].

## Method

### Participants

39 children and adolescents aged 9–16 years (*M* = 13.8; *SD* = 2.2) and their parents participated in the study. Young people with OCD were recruited via referrals from an OCD outpatient clinic and other community mental health organizations for children and adolescents. OCD group participants were invited to receive 12 sessions of FCBT for OCD [45, 46] provided by one of two registered clinical psychologists at either a child community mental health clinic or a university psychology clinic. Control participants, recruited through advertising via university research participation sites and databases, received two movie tickets to thank them for participation in the study. Children and adolescents who met diagnostic criteria for OCD were allocated to the OCD group, and young people who did not meet criteria for any clinical disorders were assigned to the non-clinical control group. The OCD group included 19 children and adolescents (47% female, n = 9) with a mean age of 14.5 (*SD* = 2.0) years. All participants in this group had a principal diagnosis of OCD based on the most recent interview schedule available for children and adolescents at the time of testing, the Anxiety Disorders Interview Schedule for DSM-IV—Child/Parent Versions (ADIS-IV-C/P; [47]). All OCD group participants also met Diagnostic and Statistical Manual of Mental Disorders, Fifth Edition, Text Revision (DSM-5-TR, [5]) diagnostic criteria for OCD. The mean clinician severity rating (CSR; ADIS-IV, [47]) for participants’ OCD was 6.0 (*SD* = 0.9) on a 0 to 8 scale, where higher scores indicate greater severity and interference. Most OCD group participants also met criteria for a secondary (less severe) comorbid diagnosis (58%, n = 11), including generalized anxiety disorder (GAD, 21%), social anxiety disorder (16%), ADHD (11%), separation anxiety disorder (5%), and specific phobia (5%). The control group comprised 20 children and adolescents (45% female, n = 9) with a mean age of 13.2 (*SD* = 2.2) years who did not meet criteria for any clinical disorders assessed by the ADIS-IV-C/P.

The two groups were compared based on age, gender, and other socio-demographic variables using analysis of variance (ANOVA) and Chi-square tests, as appropriate. No significant group differences were found for child mean age (*F* (1, 39) = 3.77, *p* = .06), child gender (*χ2* (1, N = 39) = 0.02, *p* = .88), gender of attending parent (*χ2* (1, N = 39) = 3.40, *p* = .07), or other socio-demographic variables, such as number of siblings, family income level, parents’ marital status, and parents’ identified ethnicity (all non-significant *p*-values ranged from .06 to 1.0). The majority of OCD group families did not identify with a particular ethnic group (81%, n = 26), described parents’ marital status as “married/de facto” (94%, n = 30), and reported a household income of $80 000 or more per annum (79%, n = 11). OCD group families also reported a mean number of 1.2 (SD = 0.6) siblings. Similarly, the majority of non-clinical control families did not identify with a particular ethnic group (83%, n = 33), defined parents’ marital status as “married/de facto” (93%, n = 37), and reported an annual household income of $80 000 or more (85%, n = 17). Control families reported a mean number of 1.8 (SD = 0.6) siblings. Exclusion criteria for the study included comorbid diagnoses of bipolar disorder, Tourette’s disorder, pervasive developmental disorders, psychosis, and any other conditions contraindicated for the treatment intervention used and/or that could impair understanding of treatment or assessment measures (e.g., intellectual/cognitive impairments). Our sample size was comparable to, or larger than, other published studies (e.g., [26, 41–43]).

### Design

The present study employed a 2 × (3) study design with two groups (i.e., OCD and non-clinical control groups) and three parent–child discussion tasks about (a) pleasant, (b) anxiety-provoking, and (c) conflict, situations. OCD and non-clinical control groups were compared based on self-report measures and observational data obtained from the three parent–child discussion tasks. OCD group participants who received treatment for OCD completed the discussion tasks and self-report measures again after completing FCBT for OCD [45, 46]. Thus, pre- and post-treatment data were compared using a within-subjects repeated-measures design. The current study was approved by The University of Sydney Human Research Ethics Committee (project numbers: 2014/462; 2015/571) and Sydney Local Health District Human Research Ethics Committee (project numbers: HREC/11/CRGH/277; HREC/17/CRGH/116).

### Measures

#### Anxiety Disorders Interview Schedule for DSM-IV – Child/Parent Version (ADIS-IV-C/P; [47])

The clinician-administered semi-structured interview is used to diagnose anxiety and related disorders in children and adolescents using DSM-IV criteria. The ADIS-IV was the most recent interview schedule available for young people at the time of testing. The ADIS-IV-C/P has excellent psychometric properties and has commonly been used in anxiety research with young people [48]. A clinician severity rating (CSR) of ≥ 4, on a scale ranging from 0 to 8, indicates a disorder of clinical significance. Inter-rater reliability for principal diagnoses was calculated for 25% of participants from the OCD and control groups, randomly selected using computer-generated random numbers. An independent evaluator (IE), a doctoral level clinical psychologist, reviewed video recordings of both child and parent ADIS-IV interviews for the randomly selected participant sample. Excellent inter-rater reliability was obtained, evidenced by an agreement rate of 100 percent (κ = 1.0) for principal diagnosis for both participant groups.

#### Children’s Yale-Brown Obsessive Compulsive Scale (CY-BOCS)—Child and Parent Report (PR)

The CY-BOCS [49] semi-structured and clinician-rated interview assesses OCD symptom severity in children and adolescents, aged 6 to 17 years. The 10-item measure evaluates obsessions and compulsions using a 5-point Likert scale. Total scores indicate subclinical (0–7), mild (8–15), moderate (16–23), severe (23–31), or extreme (32–40), OCD symptoms [50]. The CY-BOCS has demonstrated good psychometrics, including validity and reliability [49]. Internal consistency for the current sample was good (α=0.83). The CY-BOCS-PR [18], based on the original CY-BOCS interview, is a 10-item measure of child OCD symptom severity rated by parents. Psychometric properties for the CY-BOCS-PR are adequate [18]. Internal consistency was fair for the current sample (α=0.72).

#### McMaster Family Assessment Device (FAD; [51])

The FAD assesses family functioning according to six domains: communication, problem-solving, affective involvement, affective responsiveness, roles, and behavior control. The self-report measure uses a 4-point scale, with lower scores indicating higher levels of healthy family functioning. The general functioning (GF) scale offers a summary of general family functioning using 12 items, with a mean score of less than 2 denoting healthy family functioning. The FAD has demonstrated sound reliability and has been able to significantly distinguish “healthy” from “unhealthy” clinic-rated families [51, 52]. Internal consistency for the current sample was good for child report (α=0.88 and excellent for parent report (α=0.90).

#### Pediatric Accommodation Scale – Parent Report (PAS-PR; [53])

The PAS-PR is a 10-item parent-report measure assessing the frequency and severity/interference of accommodation behavior relating to anxiety. The measure comprises three subscales: Frequency (frequency of accommodation behavior), Parent Impact Scale (degree to which accommodation behaviors have impacted the parent/family), and Child Impact Scale (degree to which accommodation impacted the child’s functioning at school, with friends, or at home with family). The measure uses a 0–4 scale, where higher scores indicate increased frequency or severity/interference. The measure has demonstrated good internal consistency and excellent inter-rater reliability [53]. Internal consistency for the current sample was good to excellent (α=0.94 Frequency Scale; α=0.86 Parent Impact Scale; α=0.94 Child Impact Scale).

#### Spence Children’s Anxiety Scale – Child and Parent Report (SCAS – C/P; Spence [54])

The SCAS-C/P is a self-report measure assessing anxiety symptoms in children and adolescents aged 8 to 17 years. The measure assesses anxiety using six subscales associated with common anxiety disorders (e.g., separation anxiety, social anxiety, generalized anxiety, obsessions/compulsions) and/or provides a total score. Items are rated on a 0–3 scale, where higher scores denote elevated levels of anxiety symptoms [54]. Total scores for child and parent report have shown excellent reliability [55, 56]. The measure has good retest reliability and internal consistency and shows good discrimination between anxious and non-anxious children [57]. Internal consistency for total score for the current sample was good for child report (α=0.88) and excellent for parent report (α=0.96).

#### Children’s Depression Inventory – Short Form (CDI-S; [58])

The CDI-S assesses symptoms of depression in children and adolescents aged 7–17 years using 10 self-report items. Each item is rated on a 0–2 scale, with higher scores signifying elevated levels of depressive symptoms. The CDI-S has shown comparable results to the 27-item long-form CDI, *r* = 0.89 [58]. The short-form measure has also shown acceptable psychometrics, including reliability [58, 59]. Internal consistency for the current sample was good (α=0.87).

#### Depression Anxiety Stress Scales-21 (DASS-21; [60]

The DASS-21 assesses the severity and frequency of adults’ depression, anxiety, and stress symptoms over the past week using 21 self-report items rated on a 4-point scale. Total scores are calculated for the depression, anxiety, and stress scales respectively and fall within the range of *normal* to *extremely severe*. The measure has demonstrated good reliability and validity [61]. Internal consistency for the current sample was good for depression (α=0.88), anxiety (α=0.87) and stress (α = 0.81) scales.

#### Observed Behavioral Dimensions

Parent and child behaviors observed from the video-recorded discussions were coded based on the dimensions of warmth, aversiveness, withdrawal, confidence, doubt, positive problem-solving, reassurance, over involvement (parent only), autonomy granting (parent only), and responsibility enhancement (own or other). Refer to Table 1 for definitions of behavioral dimensions. Coding was based on the macro-coding schedule by Mathieu et al. [43], shown to have sound inter-rater reliability (ranging from moderate to excellent), modified in the current study to include two additional variables from the extant literature, doubt and problem-solving. Items were rated on a unidirectional 4-point Likert scale, ranging from 0 (none at all) to 3 (extreme). Whether or not a solution or agreement was reached during the discussion (0 = no,1 = yes) and the person responsible for implementing the solution (0 = child; 1 = parent) were also rated (relevant for conflict and anxiety discussions only). Overall quality of parent–child interactions was rated on a 5-point Likert scale, ranging from 0 (very poor) to 4 (excellent). Each recording was viewed at least twice to complete coding: (1) to code parent items, (2) to code child items. The 5-min recording was paused at 1-min intervals to rate each child/parent behavioral dimension on their respective scales. A mean score was later calculated for each dimension. After watching the video at least twice, overall or general items of quality of interaction and responsibility enhancement were coded based on the entire 5-min discussion. To establish reliable coding, two raters, registered clinical psychologists, were trained in the use of the coding schedule. This was achieved by practicing on a small sample of videos until substantial agreement (i.e., 0.60 or greater; [42, 43, 62]) for each dimension was obtained. Following training, one rater viewed and coded video recordings for the entire sample. The second rater, blind to group condition, coded 20% of the total sample’s video recordings (randomly selected using computer-generated random numbers) to obtain inter-rater reliability for each parent and child behavioral dimension. Intraclass correlations of mean scores for behavioral dimensions are presented in Table 2 for each of the discussion tasks. In a few instances, a behavioral dimension (e.g., aversiveness, withdrawal, reassurance seeking/providing) from one or more of the tasks did not show sufficient variation in the data (i.e., both raters scored below 1) for interrater reliability analyses to be applicable. Overall, correlations showed good to excellent reliability [62, 63], with the majority of correlations also showing significance.

**Table 1:** Definitions of parent and child behavioral dimensions for coding

Behavioral dimension	Definition	Scoring
Warmth	Expressing positive regard for other, e.g., showing: affection, reciprocity of feelings, support, encouragement	Minute-by-minute
Aversiveness	Criticism or hostility toward other	Minute-by-minute
Withdrawal	Lack of engagement with task and/or other, including lack of interest or participation, lack of emotional reciprocity or support	Minute-by-minute
Confidence	Expressing or demonstrating belief in their/other's ability to deal with situation or solve the problem	Minute-by-minute
Doubt	Questionning own/other's ability to complete the task successfully or implement the solution	Minute-by-minute
Positive problem-solving	Facilitating or suggesting positive and assertive approaches to deal with the problem	Minute-by-minute
Reassurance seeking/providing	Reassurance sought by child from parent or reassurance given to child by parent about the task or situation being discussed	Minute-by-minute
Overinvolvement (parent only)	Excessive restriction of child and encouragement of dependence, interfering with age-appropriate levels of autonomy	Minute-by-minute
Autonomy granting (parent only)	Acknowledging and encouraging child's ideas, opinions, and choices; seeking their input on problem-solving/decision making	Minute-by-minute
Responsibility child/parent enhance own	Degree to which child/parent enhances their own responsibility during the problem-solving task	Overall
Responsibility child/parent enhance other	Degree to which child/parent enhances the other's responsibility during the problem-solving task	Overall

**Table 2:** Inter-rater reliability: intra-class correlation coefficients (ICC) and significance for behavioral dimensions

Behavioral dimension	Pleasant		Anxiety		Conflict	
	ICC	*p*	ICC	*p*	ICC	*p*
Child behaviors						
Warmth	0.98	< .001	0.91	.003	0.92	.002
Aversiveness	*b*	*b*	*b*	*b*	*b*	*b*
Withdrawal	0.75	.044	*b*	*b*	0.89	.005
Confidence^a^	n/a	n/a	0.85	.011	0.98	< .001
Reassurance seeking	*b*	*b*	0.99	< .001	*b*	*b*
Doubt^a^	*n/a*	*n/a*	0.92	.002	0.92	.008
Problem-solving^a^	*n/a*	*n/a*	0.94	< .001	1.00	< .001
Parent behaviors						
Overinvolvement	0.91	.003	0.94	< .001	1.00	
Autonomy granting	0.91	.003	0.88	.006	0.90	.003
Warmth	0.99	< .001	0.93	.001	0.91	.003
Aversiveness	*b*	*b*	*b*	*b*	1.00	
Withdrawal	*b*	*b*	0.89	.005	1.00	
Confidence^a^	n/a	n/a	0.76	.041	0.60	.145
Reassurance providing	*b*	*b*	*b*	*b*	*b*	*b*
Doubt^a^	n/a	n/a	0.98	< .001	0.83	.017
Problem-solving^a^	n/a	n/a	0.86	.009	0.96	< .001

^a^behavior dimension not relevant to pleasant situation discussion, *n/a* not applicable

^b^correlation coefficient not calculated due to zero variance items

### Procedure

Following written informed consent, children and adolescents and their parents were interviewed separately by registered psychologists/clinical psychologists using the ADIS-IV-C/P [47] to determine diagnostic status and suitability for the study. Young people who met principal diagnostic criteria for OCD based on child and parent interviews were allocated to the OCD group, while young people who met criteria for no clinical disorders were allocated to the control group. Young people and their parents from both groups completed self-report measures assessing family factors and symptoms of anxiety and depression. Parent–child dyads then participated in three 5-min discussion tasks about (a) a pleasant situation, (b) an anxiety-provoking situation, and (c) a conflict situation, that were each video-recorded. Participants were asked to discuss: (a) “a time you enjoyed”, (b) “something often argued about at home”, and (c) “a situation the young person finds anxiety-provoking”, respectively. Counterbalancing was used for the tasks: participants were randomly assigned to complete the discussions in one of six order combinations based on computer-generated random numbers. Parent–child dyads were asked to select a topic for each of the three discussions that was relevant to their family. Participants could choose a discussion situation from the respective list of topics or nominate their own topic if more applicable. Examples of the idiosyncratic topics included: “a family holiday” for the pleasant situation, “a class test/exam” for the anxiety-provoking situation, and “staying up late” for the conflict situation. Dyads were asked to talk about the nominated situation for five minutes and “discuss the young person’s feelings and behaviors in the situation”. In the anxiety and conflict situation discussions, dyads were also asked to “try to reach agreement or solve the problem” (instructions modified from [64]). Once the topic was selected, the researcher left the room for the duration of the video-recorded discussion, returning after 5 min. A proportion of the OCD group completed self-report measures and participated in the discussion tasks again (using a new topic for each of the repeated discussions) after participating in FCBT for OCD [45, 46], allowing for pre-post comparisons.

### Treatment

Treatment involved a FCBT intervention for OCD comprising both child- [46] and family-focused [45] components. The child-focused intervention included 12 one-hour sessions involving ERP and cognitive restructuring. The family-focused intervention consisted of 6 one-hour sessions, held after alternate child sessions, targeting family factors such as FA, blame, conflict, cohesion, and problem-solving. It was a requirement for family sessions that at least one parent/caregiver attend all sessions with the young person, however, the whole family was encouraged to attend where possible. One of two registered clinical psychologists facilitated all treatment sessions for each young person and their family. The FCBT intervention employed has been evaluated previously, including in two randomized controlled trials, and shown excellent outcomes[32, 45, 65].

### Statistical Analyses

Statistical analyses were conducted using IBM SPSS Statistics for Windows, Version 28.0 (IBM [66]). Cohen’s kappa coefficient was calculated to determine agreement between two independent raters for principal Axis I diagnoses using DSM-IV diagnostic criteria assessed by the ADIS-IV. Demographic data for the two participant groups were compared using one-way ANOVAs and Chi-square tests, as applicable. Group comparisons of child/parent self-report measures for OCD and control groups were conducted using one-way ANOVAs. Child/parent self-report measures for OCD group participants who completed FCBT for OCD were also compared pre- and post-treatment using repeated measures ANOVAs. Completion of a minimum of 80% of questionnaire items was required for analyses, with mean scores replacing any missing items. Observer ratings of child/parent behavior by two independent raters were compared using intraclass correlations to ascertain inter-rater reliability. Multivariate analyses of variance (MANOVAs) were conducted for each of the three parent–child discussion situations (i.e., pleasant, anxiety-provoking, and conflict) to assess group differences for observer-rated child and parent behavioral dimensions, respectively. The two groups were compared using Chi-square tests on clarity of solution (whether or not dyads reached a clear solution) and responsibility for the solution (whether the parent or child took responsibility for the problem solution) for the anxiety and conflict discussions. Group comparisons using one-way ANOVAs were conducted for quality of parent–child interactions for each of the three discussions. Exploratory analyses, using repeated measures ANOVAs, were used to compare observer-rated parent/child behavioral dimensions pre- and post-treatment for OCD group participants who completed FCBT for OCD. These exploratory analyses were only conducted on variables showing significant effects in the MANOVA group comparisons for each respective discussion task. Pre-post comparisons using McNemar tests [67] were also conducted for clarity of solution and responsibility for solution for the relevant anxiety and conflict discussions. Quality of parent–child interactions were compared pre- and post-treatment using repeated measures ANOVAs. Relationships between pre-post changes in observed parent/child behaviors and OCD symptom change were investigated using Pearson Correlation Coefficients. Pearson Correlation Coefficients were calculated based on change scores computed for the CY-BOCS and each relevant parent and child behavioral dimension.

## Results

### Child and Parent Self-Report Measures

#### Group Comparisons

Table 3 presents the means and standard deviations for child and parent self-report symptom and family measures for OCD and control groups. Missing self-report data was minimal; one SCAS-C, one FAD-C, and two PAS-PR questionnaires were not completed, and another participant failed to complete any self-report questionnaires. One-way ANOVAs were used to compare groups on each measure. *F* values and related significance are also reported in Table 3. The OCD group demonstrated significantly higher scores than the control group on symptom measures, including parent/child-rated SCAS (*p’s* < .001), CDI (*p* = .001), and the DASS-21-Depression scale (*p* < .02), but not on the DASS-21-Anxiety/Stress scales. The OCD group also demonstrated significantly higher scores compared to the control group on parent-rated family measures, including FAD-GF (*p* = .004) and PAS-Frequency/Child Impact/Parent Impact scores (all *p’s* < .001), but not on the child-rated family measure, FAD-GF.

**Table 3:** Parent and child symptom and family measures

a. Means and standard deviations for comparisons of OCD and control groups			
Measures	OCD *M* (SD)	Controls *M* (SD)	*F (sig)*
Child report			
SCAS	39.8 (14.0)	18.8 (8.9)	30.41***
CDI	5.2 (5.1)	1.1 (1.5)	12.26***
FAD-GF	1.9 (0.6)	1.6 (0.4)	1.91
Parent report			
SCAS	42.8 (13.7)	12.2 (9.5)	65.10***
FAD-GF	2.0 (0.6)	1.5 (0.4)	9.61**
PAS-Frequency	10.8 (5.1)	3 (3.8)	27.10***
PAS-Child Impact	5.5 (3.3)	1.1 (1.8)	24.63***
PAS-Parent Impact	3.4 (2.0)	0.7 (1.1)	24.21***
DASS-21-Depression	8.1 (7.5)	2.9 (4.9)	6.52*
DASS-21-Anxiety	2.9 (4.4)	2.7 (6.1)	0.02
DASS-21-Stress	12.2 (7.0)	8.7 (7.5)	2.16

b. Pre-post treatment comparisons for OCD group			
Measures	Pre *M* (SD)	Post *M* (SD)	*F (sig)*
Clinician report			
ADIS-IV CSR	5.9 (0.8)	3.3 (1.4)	46.20***
CY-BOCS	23.0 (6.6)	13.8 (8.4)	30.75***
Child report			
SCAS	42.5 (13.5)	32.5 (13.8)	18.40***
CDI	5.0 (4.1)	3.1 (3.7)	4.70*
FAD-GF	1.8 (0.6)	1.7 (0.6)	1.33
Parent report			
CY-BOCS	26.5 (4.6)	14.0 (8.6)	85.55***
SCAS	43.8 (15.4)	29.8 (14.5)	19.5***
PAS-Frequency	10.6 (4.7)	5.7 (4.1)	36.04***
PAS-Child Impact	5.2 (3.0)	2.3 (2.5)	26.71***
PAS-Parent Impact	3.1 (1.9)	1.6 (1.7)	25.19***
FAD-GF	2.1 (0.6)	2.0 (0.7)	0.51

*M* mean, *SD* standard deviation,* F* F-test, *sig* significance

^*^p ≤ .05, **p ≤ .01, ***p ≤ .001

#### Pre-Post Comparisons

Table 3 presents means and standard deviations for clinician-rated measures and child/parent self-report measures completed pre- and post-treatment for the 13 OCD group participants who completed FCBT for OCD. Missing data was minimal and included one CY-BOCS-PR and one FAD-C/P questionnaire. Repeated measures ANOVAs were used to compare pre- and post-treatment scores. *F* values and associated significance are also reported in Table 3. There were significant pre- to post-treatment improvements in scores for the ADIS-IV CSR, CY-BOCS, CY-BOCS-PR, CDI, child- and parent-rated SCAS-Total Score, and PAS-Frequency/Child Impact/Parent Impact, all *p*’s < .05. No significant treatment effects were found for child- and parent-rated FAD-GF.

### Observation of Child and Parent Behaviors

#### Group Comparisons

MANOVAs were conducted for each parent–child discussion task to assess group differences in observer-rated child and parent behaviors, respectively. Table 4 presents means, standard deviations, *F* values and associated significance for child and parent variables included in MANOVAs for each of the three discussions.

**Table 4:** Observed child and parent behaviors

a. Comparisons of means and standard deviations for OCD and control groups									
Behavioral dimension	Pleasant			Anxiety			Conflict		
	OCD *M* (SD)	Controls *M* (SD)	*F (sig)*	OCD *M* (SD)	Controls *M* (SD)	*F (sig)*	OCD *M* (SD)	Controls *M* (SD)	*F (sig)*
Child behaviors									
Warmth	1.7 (0.7)	2.4 (0.6)	12.24***	1.6 (0.6)	2.0 (0.5)	7.20**	1.5 (0.6)	2.1 (0.6)	7.86**
Aversiveness	0.0 (0.0)	0.0 (0.0)		0.0 (0.0)	0.0 (0.1)	1.69	0.0 (0.0)	0.1 (0.3)	0.23
Withdrawal	0.2 (0.4)	0.0 (0.0)	3.83	0.2 (0.4)	0.0 (0.0)	3.10	0.2 (0.5)	0.0 (0.0)	3.00
Confidence^a^	n/a	n/a	n/a	1.1 (0.7)	2.1 (0.8)	25.00***	1.9 (1.1)	2.0 (0.7)	0.25
Doubt^a^	n/a	n/a	n/a	1.6 (0.7)	0.6 (0.5)	23.39***	0.9 (1.0)	0.4 (0.5)	3.07
Reassurance seeking	0.0 (0.1)	0.0 (0.0)	1.18	0.1 (0.2)	0.0 (0.1)	0.72	0.0 (0.0)	0.0 (0.0)	
Problem-solving^a^	n/a	n/a	n/a	1.5 (0.6)	2.1 (0.6)	11.87***	1.4 (0.8)	1.8 (0.5)	2.17
Responsibility child enhance own^a^	n/a	n/a	n/a	0.1 (0.2)	0.7 (0.7)	15.49***	0.2 (0.6)	0.6 (0.5)	4.91*
Responsibility child enhance parent^a^	n/a	n/a	n/a	0.1 (0.3)	0.2 (0.4)	0.12	0.3 (0.5)	0.4 (0.8)	0.31
Parent behaviors									
Overinvolvement	0.1 (0.2)	0.0 (0.0)	1.79	0.1 (0.3)	0.0 (0.1)	2.20	0.0 (0.1)	0.1 (0.2)	0.41
Autonomy granting	2.2 (0.6)	2.2 (0.5)	0.13	2.3 (0.7)	2.4 (0.6)	0.18	2.0 (0.7)	1.9 (0.7)	0.33
Warmth	2.2 (0.5)	2.4 (0.6)	1.75	1.8 (0.4)	2.2 (0.6)	4.45*	1.8 (0.5)	2.1 (0.7)	3.18
Aversiveness	0.0 (0.0)	0.0 (0.0)		0.0 (0.1)	0.0 (0.1)	0.01	0.0 (0.1)	0.0 (0.0)	2.52
Withdrawal	0.0 (0.0)	0.0 (0.1)	0.85	0.0 (0.1)	0.0 (0.0)	1.12	0.0 (0.1)	0.0 (0.2)	0.40
Confidence^a^	n/a	n/a	n/a	1.7 (0.5)	2.3 (0.6)	12.44***	1.9 (0.4)	2.0 (0.6)	0.08
Doubt^a^	n/a	n/a	n/a	0.6 (0.6)	0.2 (0.4)	4.62*	0.6 (0.7)	0.4 (0.5)	1.46
Reassurance providing	0.0 (0.0)	0.0 (0.0)		0.0 (0.1)	0.1 (0.2)	0.81	0.0 (0.1)	0.0 (0.0)	1.18
Problem-solving^a^	n/a	n/a	n/a	1.8 (0.6)	2.3 (0.7)	4.54*	1.7 (0.7)	1.9 (0.7)	0.73
Responsibility parent enhance own^a^	n/a	n/a	n/a	0.8 (0.9)	0.2 (0.4)	6.98*	0.5 (0.6)	0.6 (0.7)	0.22
Responsibility parent enhance child^a^	n/a	n/a	n/a	0.5 (0.6)	0.8 (0.6)	1.74	0.6 (0.5)	1.0 (0.6)	5.02*

b. Pre-post treatment comparisons for OCD group									
Behavioral dimension	Pleasant			Anxiety			Conflict		
	Pre *M* (SD)	Post *M* (SD)	*F (sig)*	Pre *M* (SD)	Post *M* (SD)	*F (sig)*	Pre *M* (SD)	Post *M* (SD)	*F (sig)*
Child behaviors									
Warmth	1.9 (0.6)	2.0 (0.6)	1.37	1.7 (0.4)	1.7 (0.4)	0.32	1.7 (0.5)	1.7 (0.5)	0.51
Confidence				1.2 (0.7)	1.6 (0.6)	5.57*			
Doubt				1.7 (0.7)	1.4 (0.8)	4.95*			
Problem-solving^a^				1.7 (0.5)	1.5 (0.4)	1.21			
Responsibility child enhance own^a^				0.1 (0.3)	0.2 (0.4)	0.32	0.3 (0.6)	0.3 (0.5)	0.00
Parent behaviors									
Warmth				1.8 (0.4)	1.8 (0.4)	0.05			
Confidence^a^				1.8 (0.5)	2.0 (0.7)	1.70			
Doubt^a^				0.5 (0.6)	0.4 (0.6)	0.12			
Problem-solving^a^				1.8 (0.6)	1.6 (0.3)	1.18			
Responsibility parent enhance own^a^				0.8 (0.7)	0.1 (0.3)	11.05**			
Responsibility parent enhance child^a^							0.7 (0.5)	0.7 (0.5)	0.00

*M* mean, *SD* standard deviation,* F* F-test, *sig* significance, *a* behavior dimension not relevant to pleasant situation discussion, *n/a* not applicable

^*^p ≤ .05, **p ≤ .01, ***p ≤ .001

#### Child Behavior

Refer to Table 4. *Pleasant Situation Discussion.* The MANOVA assessing group differences in child behavior observed during the pleasant situation discussion was significant, *F*(3,33) = 4.45, *p* = .01. Follow-up tests showed significantly lower scores for the OCD group compared to the control group on warmth (*p* = .001). Groups did not differ significantly on reassurance seeking (*p* = .28) or withdrawal (*p* = .06) for this discussion. *Anxiety-Provoking Situation Discussion.* The MANOVA assessing group differences for the anxiety discussion was significant, *F*(9, 28) = 6.32, *p* < .001. Follow-up tests indicated significantly higher scores for the OCD group on doubt (*p* < .001), and significantly lower scores on warmth (*p* = .01), positive problem-solving (*p* = .001), confidence (*p* < .001), and child enhancing own responsibility (*p* < .001), compared to the control group. No significant group differences were evident for aversiveness (*p* = .20), withdrawal (*p* = .09), reassurance seeking (*p* = .40), and child enhancing parents’ responsibility (*p* = .73). *Conflict Situation Discussion.* The MANOVA conducted for the conflict discussion showed a non-significant overall effect of group, *F*(8, 28) = 2.19, *p* = .06. Although the main effect only approached significance, it is notable that the OCD group scored significantly lower than the control group on warmth (*p* < .01) and child enhancing own responsibility (*p* < .05), in individual ANOVAs.

#### Parent Behavior

Refer to Table 4. *Pleasant Situation Discussion.* The MANOVA assessing group differences in observer-rated parent behavior during the pleasant situation discussion was not significant, *F*(4, 32) = 1.76, *p* = .16. *Anxiety-provoking Situation Discussion.* The MANOVA assessing group differences in observed parent behavior during the anxiety discussion was significant, *F*(11, 26) = 2.73, *p* = .02. Follow-up tests indicated significantly lower scores for parents in the OCD group on warmth (*p* = .04), confidence (*p* = .001), and positive problem-solving (*p* = .04), compared to controls. The OCD group also showed significantly higher scores on doubt (*p* = .04) and parents enhancing their own responsibility (*p* = .01). *Conflict Situation Discussion.* The MANOVA assessing group differences during the conflict situation discussion showed an overall significant effect of group, *F*(11, 25) = 2.49, *p* = .03. Follow-up effects showed significantly lower scores for the OCD group on parents enhancing child responsibility (*p* = .05).

*Clarity of Solution*. *Anxiety-provoking Situation Discussion*. During the anxiety discussion, only 9 parent–child dyads reached a clear solution. Of these, 33% were OCD dyads and 67% control dyads. Twenty-nine dyads did not reach a clear solution: 52% were from the OCD group and 48% from the control group. No significant differences between groups were evident on whether a clear solution was reached or not, *χ2* (1, N = 38) = 0.93, *p* = .33. *Conflict Situation Discussion.* During the conflict discussion, 13 dyads reached a clear solution: 38.5% were OCD and 61.5% were control dyads. Twenty-five dyads did not reach a clear solution: 52% were OCD and 48% control dyads. There was no significant group difference on whether a clear solution was reached, *χ2* (1, N = 38) = 0.63, *p* = .43.

*Responsibility for Solution*.* Anxiety-provoking Situation Discussion*. During the anxiety discussion, 26 young people were identified as responsible for generating the solution or implementing the final agreed-upon solution: 27% from the OCD and 73% from the control group. Eight parents were identified as responsible for generating or implementing the solution: 100% were parents from the OCD group. There was a significant difference between groups on whether the child or parent took responsibility for the solution, *χ2* (1, N = 39) = 13.25, *p* < .001. *Conflict Situation Discussion.* During the conflict discussion, 22 young people were responsible for the solution: 36% were from the OCD group and 63% were controls. Thirteen parents were identified as responsible for the solution: 54% OCD group participants and 46% controls. No significant group differences were evident on whether the parent or child took responsibility for the solution, *χ2* (1, N = 35) = 13.25, *p* < .001.

*Quality of Interaction*. Group differences in overall quality of parent–child interactions during each of the three discussions were assessed. Significant group differences in interaction quality were evident for the pleasant situation discussion, *F*(1, 38) = 2.22, *p* = .05, and anxiety-provoking situation discussion, *F*(1, 38) = 2.11, *p* = .01, with OCD group dyads demonstrating poorer quality interactions compared to control dyads. No significant group differences on quality of interaction were evident during the conflict situation discussion, *F*(1, 38) = 0.12, *p* = .68.

#### Pre-Post Comparisons

Repeated measures ANOVAs compared observer-rated child and parent behaviors pre- and post-treatment for the 13 OCD dyads who completed FCBT for OCD. The exploratory analyses were conducted on variables showing significant effects in the group comparisons above for each discussion. Results are presented in Table 4.

#### Child Behavior

Refer to Table 4*. Pleasant Situation Discussion. *Warmth did not show significant change pre- to post-treatment, *F*(1, 12) = 1.37, *p* = .27. *Anxiety-provoking Situation Discussion*. Confidence (*F*(1, 12) = 5.57, *p* = .04) and doubt (*F*(1, 12) = 4.95, *p* = .05) showed significant improvements pre- to post-treatment. Warmth, problem-solving, and child enhancing own responsibility did not show significant treatment effects (*p*’s > .06). *Conflict Situation Discussion.* Warmth (*F*(1, 12) = 0.51, *p* = .49) and child enhancing own responsibility (*F*(1, 12) = 0.00, *p* = 1.0) did not show significant treatment effects.

#### Parent Behavior

Refer to Table 4*. Anxiety-provoking Situation Discussion*. Parent enhancing own responsibility showed a significant pre- to post-treatment reduction (*F*(1, 12) = 11.05, *p* < .01). Warmth, confidence, doubt, and problem-solving did not show significant treatment effects (*p’s* > .22). *Conflict Situation Discussion.* Parent enhancing child responsibility did not show significant treatment effects (*F*(1, 12) = 0.00, *p* = 1.0).

*Clarity of Solution*. *Anxiety-provoking Situation Discussion*. Of the 11 dyads who did not reach a clear solution at pre-treatment, 82% (n = 9) still failed to reach a solution at post-treatment, whereas 18% (n = 2) managed to reach a problem solution at post-treatment. Zero percent of the two dyads who generated a clear solution at pre-treatment managed to reach a clear solution at post-treatment. The proportion of dyads who identified versus did not identify a clear solution during the anxiety discussion at pre-treatment compared to post-treatment was not statistically different (*p* = 1.0). *Conflict Situation Discussion.* Of the 8 dyads who did not generate a clear solution at pre-treatment, 75% (n = 6) were still unable to generate a clear solution at post-treatment, whereas 25% (n = 2) were able to identify a problem solution at post-treatment. Of the five dyads who identified a solution at pre-treatment, 100% (n = 5) were still able to generate a solution at post-treatment. The proportion of dyads who identified versus did not identify a clear solution during the conflict discussion pre-treatment compared to post-treatment was not statistically different (*p* = .5).

*Responsibility for Solution.* *Anxiety-provoking Situation Discussion*. Of the six children who took responsibility for the solution at pre-treatment, 67% (n = 4) continued to take responsibility at post-treatment, whereas 33% (n = 2) no longer took responsibility for the solution at post-treatment and a parent took responsibility instead. Of the five parents who took responsibility for the solution at pre-treatment, 20% (n = 1) continued to take responsibility at post-treatment and 80% (n = 4) no longer took responsibility and the child took responsibility instead. The proportion of children versus parents who took responsibility for the solution at pre-treatment compared to post-treatment was not statistically different (*p* = .69). *Conflict Situation Discussion.* Of the seven children who took responsibility for the solution at pre-treatment, 86% (n = 6) continued to take responsibility for the solution post treatment, whereas 14% of children (n = 1) no longer took responsibility and a parent took responsibility instead. Of the five parents who took responsibility for the solution at pre-treatment, 40% (n = 2) continued to take responsibility at post treatment, whereas 60% (n = 3) no longer took responsibility and a child took responsibility instead. The proportion of children versus parents who took responsibility for the solution at pre-treatment compared to post-treatment was not statistically different (*p* = .63).

*Quality of Interaction*. The effect of treatment on quality of interaction for each of the three discussions was assessed. No significant change in interaction quality was evident for the pleasant situation discussion (*F* (1, 12) = 1.93, *p* = .19), the anxiety-provoking situation discussion (*F* (1, 12) = 0.32, *p* = .58), or the conflict situation discussion (*F* (1, 12) = 1.68, *p* = .22).

*Behavioral dimensions and OCD symptom change.* We examined correlations between pre-post changes in OCD symptoms and observed parent/child behaviors using Pearson Correlation Coefficients on change scores for CY-BOCS and each relevant child and parent behavioral dimension. The only significant correlation was between the CY-BOCS and child enhancing their own responsibility during the anxiety discussion (*r* = − 0.56, *p* < .05). A reduction in OCD symptoms pre- to post-treatment was significantly correlated with an increase in young people enhancing their own responsibility during the anxiety discussion.

## Discussion

Increasingly, family environment factors are being identified as relevant to the development and maintenance of OCD in children and adolescents, as well as considered an important factor in the young person’s response to treatment. However, research assessing these family factors has been sparse, and therefore, our understanding of family factors relevant to OCD in young people, and their function in the condition, remains limited. More recently, observational methods, such as parent–child interaction tasks, have been recommended to measure these family constructs more precisely and objectively. Despite such recommendations (e.g., [37]), there is a dearth of empirical research in child OCD, and findings from the few observational studies conducted are mixed. The current study aimed to address these shortcomings and extend our knowledge about the familial context of OCD in young people by uniquely comparing observed parent–child behaviors in OCD families to non-clinical families across three parent–child discussion tasks (i.e., pleasant, anxiety-provoking, and conflict situation discussions). As a further strength, the current study also compared observed parent–child behaviors pre- and post-treatment for OCD group participants who completed FCBT for OCD. We hypothesized significant differences between groups across a number of behavioral dimensions, with OCD dyads scoring higher on negative dimensions (e.g., doubt) and lower on positive dimensions (e.g., warmth) than non-clinical control families. The pattern of data largely supported task-specific hypotheses for parent–child interactions in OCD, with the exception of child warmth, which demonstrated effects in the expected direction consistently for all three discussion tasks. As predicted, treatment effects emerged for parent and child behavioral dimensions.

OCD and control groups differed significantly on numerous symptom and family self-report measures, as expected. Scores on child- and parent-rated measures of child anxiety and depression significantly differentiated groups, with higher scores calculated for the OCD group. OCD group parents also scored significantly higher than controls for self-reported depression symptoms, with scores falling in the moderate, compared to normal, range, further supporting the literature regarding elevated mental health issues in family members of young people with OCD [41, 68, 69]. Groups in the present study also differed significantly on parent-rated family measures. Parents in the OCD group reported higher levels of FA and unhealthy general family functioning compared to controls. Child-rated general family functioning, however, did not significantly differentiate groups, with scores for both groups falling within the healthy range. The significant differences between groups, shown for parent-rated, but not for child-rated, general family functioning, can be understood in one of three ways. Firstly, some of the items comprising the general family functioning scale may measure aspects related to FA. Therefore, parent-rated reduced general family functioning may reflect areas of family life where OCD group parents have modified their behavior in response to OCD as a form of FA, for example, to avoid exacerbating child distress (e.g., “We avoid discussing our fears and concerns”). Secondly, parent-rated reduced family functioning may reflect parents’ activated coping responses related to having a child with OCD. Finally, OCD group parents may have reported poorer family functioning overall due to their experience of living with a child with OCD, a mental health condition.

As predicted, significant group differences were identified for numerous child and parent behaviors observed during discussion tasks. Interestingly, child warmth was the only behavioral dimension that differentiated OCD and control groups across all three tasks. During the pleasant, anxiety-provoking, and conflict situation discussions, young people in the OCD group demonstrated significantly lower levels of warmth towards their parents compared to controls. These findings provide support for the exploratory hypothesis predicting consistency of parent–child behaviors across discussion tasks. Lower levels of observer-rated child warmth across discussion tasks may reflect poorer family functioning, endorsed by parents, perhaps due to the stress experienced by young people and the family as a result of living with OCD. Although child warmth has not been the focus of much attention in the child OCD literature, which has concentrated more on parent behavior (e.g., parent warmth), child warmth was the only child or parent dimension that significantly differentiated OCD and non-clinical control groups in all three observational studies (i.e., [41–43]) that used the recommended unidirectional measurement scales (e.g., [37]). As child warmth was a dimension not affected by task context, low warmth demonstrated by children and adolescents with OCD may be a more stable, general way of interacting with parents, requiring further investigation. Aside from potentially reflecting reduced general family functioning and family stress related to OCD, low warmth may have occurred initially as a response to anxious affect and then generalized to other situations, or alternatively, may be affected by trait levels of emotional inhibition.

Whilst child warmth demonstrated consistency across the present study’s discussion tasks, other behavioral dimensions differentiating groups showed specificity regarding tasks, emphasizing the importance of context for particular parent–child interaction behaviors in OCD. Most of the child and parent behaviors distinguishing groups were evident only during the anxiety discussion, a task likely to evoke higher stress responses or distress, particularly for the OCD group. These behavioral dimensions included: child and parent confidence, child and parent doubt, child and parent problem-solving, and parents enhancing their own responsibility, in addition to parent warmth. Unique to the conflict discussion, the other task with the potential to evoke a stress response, parents’ enhancement of child responsibility significantly differentiated groups, such that OCD group parents enhanced child responsibility less during this discussion compared to controls. Relevant to both conflict and anxiety discussions, young people in the OCD group enhanced their own responsibility significantly less than controls. There were no significant findings specific to the pleasant situation discussion alone. Taken together, these findings provide support for the alternative exploratory hypothesis, being that significant group differences would be evident specifically during certain discussion tasks, particularly tasks evoking higher stress responses.

Contrary to our predictions, based on Farrell et al. [26] preliminary findings about responsibility enhancement in children, children and adolescents in the OCD group and their parents enhanced child responsibility significantly less overall during the relevant two problem-solving discussions (i.e., anxiety-provoking and conflict situations) than control dyads. During the anxiety discussion, OCD group parents were more likely to enhance their own responsibility (including for the problem solution) compared to controls. Children and adolescents with OCD were also less likely to take responsibility during the anxiety discussion, including for generating and implementing a solution, compared to controls. Enhanced parent responsibility, and reduced enhancement of child responsibility by OCD group parents and young people, particularly during the anxiety discussion, may be associated with FA and child avoidance behaviors common in OCD and anxiety. That is, a parent of a child with OCD may shoulder more responsibility during the anxiety discussion to reduce or prevent child distress, thereby accommodating child anxiety and related behaviors. Similarly, a child with OCD may assume less responsibility in order to avoid or reduce anxiety or distress. As the parent behavioral dimensions of overinvolvement and autonomy granting did not significantly differentiate groups in any of the three discussion tasks, it is less likely that enhanced parent responsibility (and reduced child responsibility) are aspects of these behavioral dimensions, consistent with emerging observational literature (e.g., [26, 42, 43]). Enhanced parent responsibility and reduced child responsibility are worthy of further investigation in child and adolescent samples to better understand their role in OCD symptoms and their maintenance.

Of the three discussion tasks, the anxiety-provoking situation discussion is the most comparable to other parent–child problem-solving tasks described in the literature. Overall, during this problem-solving task, as predicted, the OCD group scored significantly lower than the control group on positive behavior dimensions and higher on negative dimensions. OCD group children and adolescents showed lower levels of warmth, positive problem-solving, and confidence, and enhanced their own responsibility less compared to controls. Additionally, young people with OCD demonstrated higher levels of doubt in their ability to deal with the task and/or implement the solution compared to controls. Similar findings also emerged from observations of parent behaviors. Parents in the OCD group showed lower levels of warmth, positive problem-solving, and confidence in their child, compared to controls. OCD group parents also showed higher levels of doubt of their child and enhanced their own responsibility more, compared to controls. Parents’ elevated doubt and reduced confidence in their child may also fit an avoidance model, along with parents’ inflation of their own responsibility, as discussed above. Parents’ elevated doubt and reduced confidence are less likely to be a function of overinvolvement and aversiveness, as the latter two behavioral dimensions did not significantly distinguish groups in any of the present study’s tasks. Instead, consistent with an avoidance model, under more stressful or anxiety-provoking conditions parents may perceive their child as being less confident and less able to actively engage in problem-solving. As a consequence, parents may assume greater responsibility, and inadvertently collude with a pattern of avoidance, thereby maintaining anxiety in the longer term. The unique context in which these observed behaviors of reduced confidence, increased doubt, and increased parental responsibility occur, specifically during the anxiety discussion, provides further support for an avoidance model and interpretation of these data. Parents’ perception of their child as less capable may have been present prior to the development of OCD symptoms or may have emerged in response to OCD and the related functional impairment, with parents assuming responsibility for tasks that evoke a threat response for their child. The pattern of data obtained for young people from the anxiety discussion mirrors that of their parents. The relationship between parent and child behavior is likely to be bidirectional: a young person may be less confident, perhaps perceiving themselves as less capable, and assume less responsibility due to parents’ provision of negative coping mechanisms. Longitudinal data are necessary to rule out the explanation that the child may have always displayed these characteristics and behaviors.

The current child and parent findings are consistent with many of the results published in the observational study literature for child OCD. In particular, our findings replicate (and extend) those of Barrett et al. [41]. Where there were differences in study findings, for example discrepancies between the present findings and those of Barrett et al. [41] for doubt and autonomy granting, these were likely due to: (1) lack of topic specificity, (2) differences in task design, and/or (3) differences in coding.

Lack of topic specificity in some studies (e.g., [41]) may have contributed to discrepant findings, for example for doubt. Whereas the present study encouraged dyads to select or nominate a topic relevant to each dyad, only one topic was used for all participants in the study by Barrett et al. [41]. A generic topic may not be relevant or anxiety-provoking for some participants, possibly explaining the Barrett et al. [41] lack of significant findings for child and parent doubt. Our findings suggest that doubt may be a behavioral dimension more relevant to the maintenance of OCD than originally proposed by Barrett et al. [41].

Differences between studies in task design may have contributed to discrepancies in findings, such as those for autonomy granting [41], or problem-solving [42]. In the Barrett et al. [41] study, for example, parents were instructed that they could help their child during the problem-solving task, however, the young person was required to make the final decision. In contrast, in the present study dyads were instructed to attempt to reach agreement or solve the problem. It is likely that differences between groups for autonomy-granting would be more apparent in tasks where the parent was expected to encourage the child to make the final decision independently, compared to tasks where this directive was not provided. It is recommended that future studies consider including instructions that encourage child autonomy, so as to highlight when permitting this autonomy may be challenging for parents. Although the present study uniquely included separate discussion tasks for anxiety versus conflict situations, promoting a better understanding of the contexts in which particular child and parent behavior occurs, some studies included a mix of anxiety and conflict situations in the same discussion task, for example with the use of “hot topics” (e.g., [42, 44]). The inclusion of different valence categories in the same discussion may explain, for example, Mantz and Abbott’s [42] lack of significant findings for problem-solving, compared to our significant results, which were present only in the anxiety discussion task and not in the conflict discussion. It is recommended that future research continue to use methodologies that compare different types of negative conditions, such as threat and conflict-based conditions.

Differences in coding between studies may have also contributed to discrepant findings. Although coding in the present study was based on an established coding schedule (i.e., [43]), our results did not replicate theirs, with the exception of child warmth, and child confidence (which was approaching significance in [43]). Mathieu et al. [43] highlighted that inter-rater reliability was reduced in their final sample, and this was also comparatively lower for many behavioral dimensions compared with the present study’s good to excellent reliability. The authors also proposed that their non-significant findings may be explained by the trend that few of their parent–child dyads spoke for the full five minutes.

The present study identified significant pre- to post-treatment improvements, including on clinician-rated, parent-rated, and child-rated measures of child OCD, anxiety, and depression (e.g., ADIS CSR, CY-BOCS, SCAS, CDI). General family functioning, assessed by the FAD-GF, did not show significant changes at post-treatment according to both child and parent ratings, however scores were not particularly elevated on this measure at pre-treatment. Parent-rated FA showed a significant reduction pre- to post-treatment, as would be expected given that treatment actively targeted this unhelpful family response. Exploratory analyses investigating the effects of treatment on observed behavioral dimensions, specifically those dimensions identified as significant during group comparisons, showed some improvements pre- to post-treatment, as predicted. Treatment effects were only present for the anxiety discussion, and included child dimensions of confidence and doubt, and the parent dimension of enhancing parent responsibility. Young people’s confidence in their ability to deal with an anxiety-provoking situation and/or solve the problem increased significantly pre- to post-treatment, whilst their doubt decreased. Young people’s increased confidence and decreased doubt are somewhat unsurprising, given that treatment included exposure to feared stimuli and cognitive restructuring of anxiety-related cognitions, problem-solving practice, in addition to directly targeting unhelpful family responses, such as FA. The significant decrease pre- to post-treatment for parents enhancing their own responsibility during the anxiety discussion may be related to young people’s increased confidence and reduced doubt, impacted by the focus on unhelpful avoidance behaviors in treatment. Perhaps young people were more likely to be actively involved in the discussion and problem-solving process at post-treatment, resulting in more collaboration and/or child responsibility rather than parent responsibility being overly enhanced. As predicted, there was also a significant correlation between OCD symptom change pre- to post-treatment and family factor change, specifically young people enhancing their own responsibility during the anxiety discussion. A reduction in OCD symptoms pre- to post-treatment was significantly correlated with an increase in young people enhancing their own responsibility during the anxiety discussion, likely related to a reduction in unhelpful avoidance behaviors targeted in treatment. The remaining child and parent behavioral dimensions were not significantly correlated with OCD symptom change, consistent with findings by Schlup et al. [44], who did not assess responsibility enhancement. This suggests that the significant pre-post improvements in scores for child confidence, child doubt, and parent enhancement of their own responsibility may be more a function of the FCBT intervention and treatment process rather than a result of OCD symptom improvement. Future studies investigating the effect of treatment on observed parent–child interactions would benefit from comparing FCBT with standard CBT for OCD.

### Strengths, Limitations, and Future Directions

The present study provides an important contribution to the existing literature on observed parent–child behaviors in child and adolescent OCD. The study is the first to include three discussion task conditions, clearly distinguishing between conflict and anxiety situation topics, which have either not been included or combined in previous studies, as well as including a pleasant situation condition to assist with further comparisons. Furthermore, this is the first study to compare observed parent–child behaviors for OCD participants pre- and post-treatment for participants completing individual (rather than group) FCBT for OCD. The current study included a non-clinical comparison group of age- and gender-matched controls. All participating children and adolescents were assessed using gold-standard diagnostic interviews to determine group allocation status, including child and parent versions, both showing excellent reliability in the current sample. The current study used an established coding schedule ([43]; modified to include additional variables), with specific and unidirectional behavioral dimensions, consistent with recommendations made in the literature (e.g., [37]). In addition, inter-rater reliability in the current study was a strength, overall ranging from good to excellent, with the majority of coefficients showing significant correlations.

Overall, the study highlighted that OCD families could be differentiated from non-clinical families based on both child and parent behavior observed during parent–child interactions, in particular, during the anxiety-provoking situation discussion. Findings suggest that parent–child interaction behaviors characterizing OCD families are predominately contextual. Key child behavioral dimensions differentiating groups included: warmth, confidence, doubt, problem-solving, and responsibility enhancement. Significant parent dimensions distinguishing groups similarly included: warmth, confidence, doubt, problem-solving, and responsibility enhancement. These particular child and parent variables may be especially relevant to child and adolescent OCD, providing support for cognitive behavioral models of OCD maintenance that highlight the importance of family factors in OCD symptoms. However, this study has a cross-sectional design and therefore conclusions cannot be drawn about the direction of effects. The relationship between family factors and OCD symptoms in young people is likely to be bidirectional (e.g., [70]), with insufficient evidence to date to support a causal model for parenting and child OCD.

Although our sample size is comparable to, or larger than, other observational studies’ (e.g., [26, 41–43]), replication of the current study with a larger sample would be of benefit. Further, the significant child and parent behavioral dimensions identified above are worthy of further assessment in future studies that include a clinical control group, in particular a non-OCD, anxiety disorders group. In addition, study findings warrant replication with a less homogenous sample, including families with a range of socioeconomic status indicators, more diversity in terms of ethnicity and cultural background, and a broader range of family structures. This study presents novel data, suggesting the contextual nature of a number of parent–child interaction behaviors characterizing OCD families, laying the foundation for future research to further investigate the role and context of family factors in child and adolescent OCD.

## Data Availability

No datasets were generated or analysed during the current study.
